# Investigation of obsolete diversity of rye (*Secale cereale* L.) using multiplexed SSR fingerprinting and evaluation of agronomic traits

**DOI:** 10.1007/s13353-020-00579-z

**Published:** 2020-09-07

**Authors:** Malgorzata Targonska-Karasek, Maja Boczkowska, Wieslaw Podyma, Małgorzata Pasnik, Maciej Niedzielski, Anna Rucinska, Zuzanna Nowak-Zyczynska, Monika Rakoczy-Trojanowska

**Affiliations:** 1 Plant Breeding and Acclimatization Institute (IHAR) - National Research Institute, Radzików, Poland; 2 Polish Academy of Sciences Botanical Garden – Center for Biological Diversity Conservation in Powsin, Warszawa, Poland; 3 Department of Animal Genetics and Conservation, Warsaw University of Life Sciences, Warsaw, Poland; 4 Department of Plant Genetics, Breeding, and Biotechnology, Warsaw University of Life Sciences, Warsaw, Poland

**Keywords:** Diversity, Germplasm, Population structure, Rye, SSR

## Abstract

Common rye (*Secale cereale* L.) is one of the most important cereals in Europe. Nevertheless, its germplasm collections are among the least numerous compared with cereals. There are only about 27,000 *Secale* accessions in 70 gene banks around the world. Despite extensive research on the molecular characterization of genetic resources, only a fraction of this collection has been described. The main objective of the presented study was to perform genotypic and phenotypic characterization of an obsolete gene pool represented by 100 accessions originated from 28 countries around the world and preserved in the gene bank of the Polish Academy of Sciences Botanical Garden – Center for Biological Diversity Conservation in Powsin. Genetic analysis using simple sequence repeat markers showed that the obsolete gene pool is relatively large. This indicates that different sources of variability were used in breeding programs. However, the genetic variation is in no way related to the place of origin. Despite the great differences in the genetic make-up, the collection showed a broadly common phenotype. This could result in a low level of interest among breeders in the stored germplasm, undervalued as a source of important but not easily observable traits, e.g., high disease resistance, which was found in some accessions.

**Electronic supplementary material:**

The online version of this article (10.1007/s13353-020-00579-z) contains supplementary material, which is available to authorized users.

## Introduction

Common rye (*Secale cereale* L.) belongs to the *Poaceae* family and is one of the most important cereal crops cultivated in Europe. It is characterized by the ability to produce high yields even when grown under environmental stress conditions, i.e., low temperatures, drought, and low soil fertility. The presence of disease resistance genes reduces the need for intensive chemical protection of this crop (Korzun et al. 2001; Schlegel and Melz 1996). Moreover, rye offers high contents of many favorable compounds such as a whole suite of minerals (including Zn, Fe, and P), beta-glucans, resistant starch, and bioactive compounds. Rye products are characterized by a high level of dietary fiber (Andersson et al. 2009) that may contribute to positive health effects (Rosén et al. 2011).

A need to preserve the biodiversity of existing forms of rye and wild species as resources for plant breeders was recognized by Vavilov, who at the beginning of the twentieth century set up a collection of crops and their wild relatives, which evolved later into one of the greatest gene banks (Kulikov et al. 2013). Since then, many national, regional, and institutional gene banks have been established, most of them in the early 1970s (Knüpffer 2011). Globally, rye collections include 26,100 accessions; significantly less comparing to wheat (555,449 accessions) and barley (339,563 accessions) (FAO 2018b). Currently, genetic resources of rye are stored in 70 gene banks located in 46 countries. Because most of the species within the genus *Secale* are open-pollinated, their maintenance in gene banks is more difficult than in other cereals (such as barley and wheat). Therefore, rye ex situ collections are much smaller than wheat and barley ones, but single accessions may contain a large pool of genetic variation (Boczkowska and Puchalski 2012). Characterization of this variation contained in germplasm collections is essential for efficient gene bank management (Cruz et al. 2013). It is also crucial for the effective utilization of the available genetic resources in breeding (Lv et al. 2012).

To date, many analyses have been carried out to assess genetic diversity and phylogenetic relationships among species of the genus *Secale*, cultivars, and inbred lines. Various molecular biology methods have been used, such as RFLP (Isik et al. 2007), RAPD (Bolibok et al. 2005; Matos et al. 2001), ISSR (Bolibok et al. 2005), AFLP (Chikmawati et al. 2012; Chikmawati et al. 2005), SAMPL (Bolibok et al. 2005), SSR (Bolibok et al. 2005; Shang et al. 2006; Targońska et al. 2016), DArT (Bolibok-Brągoszewska et al. 2009; Bolibok-Brągoszewska et al. 2014), and isoenzymatic markers (Burger et al. 2006; Matos et al. 2001). Polymorphisms within the mitochondrial and chloroplast genome of rye were also analyzed (Isik et al. 2007; Skuza et al. 2007). Recently, high-throughput method genotyping by sequencing was also used in rye genetic diversity studies (Sidhu et al. 2019). The SSR method was selected in this study as a relatively cheap and sufficiently reliable tool for the basic characterization of the rye accessions.

The overall aims of this work were (i) to determine SSR based genetic diversity of obsolete gene pool of rye preserved in gene bank of Polish Academy of Sciences Botanical Garden – Center for Biological Diversity Conservation in Powsin, (ii) to evaluate the variation of agro-morphological traits, (iii) to develop a core collection, and (iv) to identify accessions with potential utility for breeding programs.

## Materials and methods

### Plant material

**Fig. 1:**
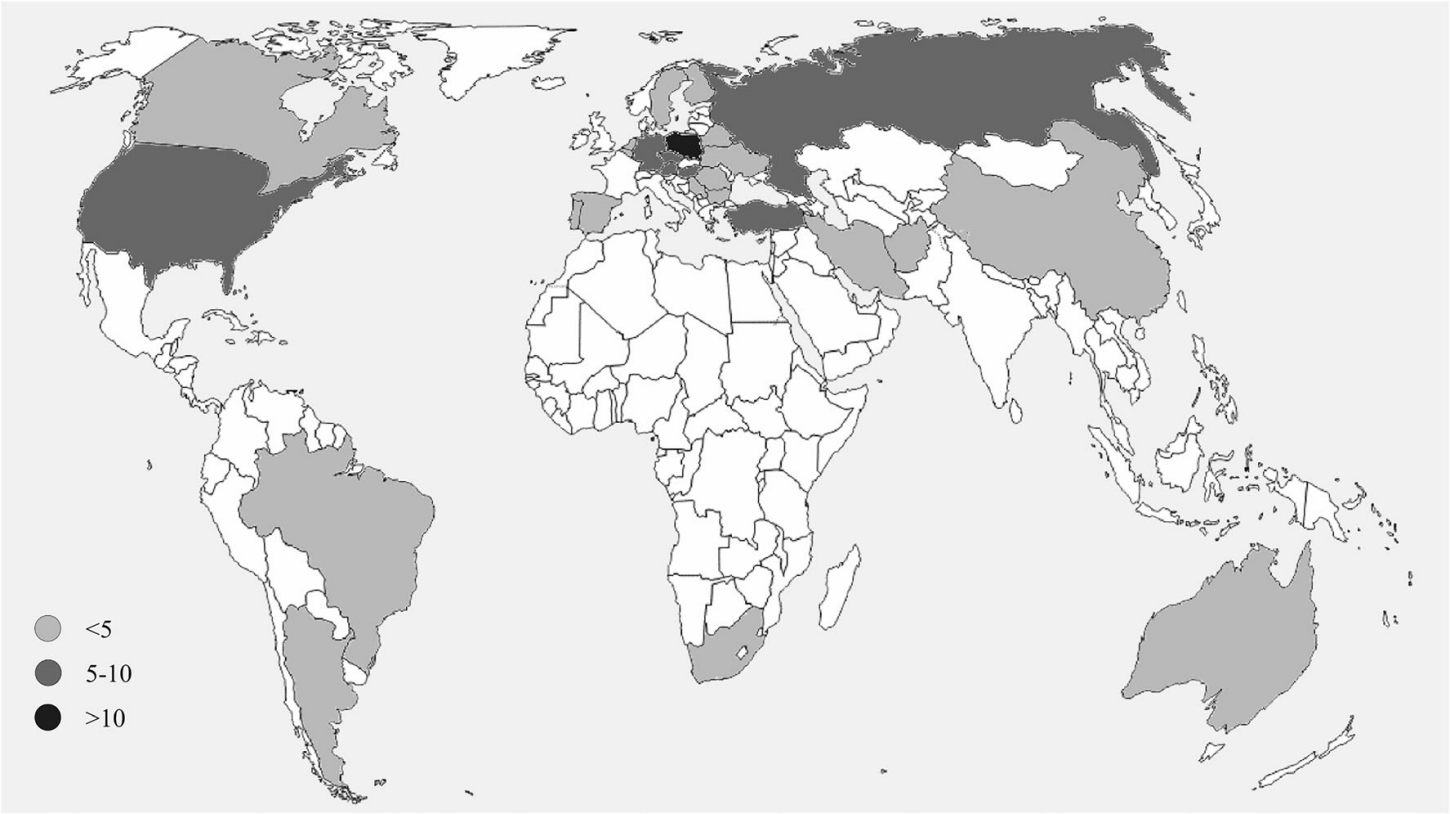
Color-coded map shows the number of accessions from each country

The plant material consisted of 100 rye accessions stored in the gene bank in the Polish Academy of Sciences Botanical Garden – Center for Biological Diversity Conservation in Powsin (PASBG). The accessions represented 28 countries (Table 1; Fig. 1) and were introduced into the collection in the years 1970–1990. Most of them were cultivars (87%), 10% were landraces, and 3% were breeding materials.

**Table 1:** Accession list containing some basic information (an asterisk indicates the accessions that have been selected for core collection)

Number	Accession number	Name	Improvement status	Acquisition year	Country of origin
1*	7261/76	683	Landrace	1976	AFG
2*	5779/75	DEBRETT	Cultivar	1975	ARG
3	8411/78	MANFREDI SUQUIA	Cultivar	1978	ARG
4	16,286/81	TERAPICO	Cultivar	1981	ARG
5	7492/76	VARNEROG	Cultivar	1976	AUS
6	1287/71	CHRYSANTH HANSERROGGENEN	Cultivar	1971	AUT
7	2525/73	HARRACH UNIVERSAL	Cultivar	1973	AUT
8*	7124/76	HOHENAUER	Cultivar	1976	AUT
9	1310/71	KARNTNER	Cultivar	1971	AUT
10	8842/79	MARCHFELDER	Cultivar	1979	AUT
11	7209/76	OTTERBACHER	Cultivar	1976	AUT
12	1276/71	SCHLAGLER	Cultivar	1971	AUT
13	1299/71	TSCHERMAKS VEREDELTER MARCHFELDER	Cultivar	1971	AUT
14	8826/79	GALMA	Cultivar	1979	BEL
15*	7910/77	JOEGEWA-AUSLESE	Cultivar	1977	BGR
16	7915/77	NISKOSTEBELNAJA	Cultivar	1977	BGR
17	2369/73	PARTIZANSKAJA	Cultivar	1973	BLR
18	6984/76	CENTENO 52	Cultivar	1976	BRA
19	7049/76	GAYEROVO	Cultivar	1976	BRA
20	7465/76	SAMPLE A	Cultivar	1976	BRA
21*	7044/76	FRONTIER	Cultivar	1976	CAN
22*	5792/75	HORTON	Cultivar	1975	CAN
23*	1198/90	SINGZHAU	Cultivar	1990	CHN
24	5780/75	DOBRENICKE KRMNE	Cultivar	1975	CSK
25	7198/76	NALZOVSKE	Cultivar	1976	CSK
26*	5831/75	VALTICKE	Cultivar	1975	CSK
27	2386/73	PUDMERICKE	Cultivar	1973	CZE
28	7403/76	ZIDLOCHOWICKIE PANIS	Cultivar	1976	CZE
29	1334/71	BENDELEBENER	Cultivar	1971	DEU
30*	18,702/83	DONAR	Cultivar	1983	DEU
31	4770/75	GULZOWER St. 1714	Cultivar	1975	DEU
32	4997/75	HESSDORFER JOHANNIS	Cultivar	1975	DEU
33*	8830/79	HGP 20	Breeding material	1979	DEU
34	18,703/83	JANOS	Cultivar	1983	DEU
35*	8840/79	LUKAS	Cultivar	1979	DEU
36*	5808/75	MECKLENBURGER MARIEN	Cultivar	1975	DEU
37*	14,982/80	PETKUSER MOORROGGEN	Cultivar	1980	DEU
38*	18,705/83	POLLUX	Cultivar	1983	DEU
39	7039/76	FLORIDA BLACK WALLANCE SELECTION	Cultivar	1976	ESP
40*	7125/76	HUESCA	Cultivar	1976	ESP
41*	7281/76	SYNTHETIC V	Cultivar	1976	ESP
42	1278/71	ENSI	Cultivar	1971	FIN
43	8833/79	HJA JUSSI 20	Cultivar	1979	FIN
44	1279/71	PEKKA	Cultivar	1971	FIN
45*	7380/76	VISA	Cultivar	1976	FIN
46	8823/79	DUNA TISZAKOZI	Cultivar	1979	HUN
47*	7036/76	FLEISCHMANN	Cultivar	1976	HUN
48*	5794/75	HUSZAJ	Cultivar	1975	HUN
49	7138/76	JAPAJEDELSKE	Cultivar	1976	HUN
50	7184/76	LOVASZPATONAI	Cultivar	1976	HUN
51	7210/76	OVARI	Cultivar	1976	HUN
52	7158/76	K 1634	Landrace	1976	IRN
53*	7012/76	DOMINANT	Cultivar	1976	NLD
54*	1295/71	DOMINANT	Cultivar	1971	NLD
55	7901/77	AR-3	Cultivar	1977	POL
56*	18,700/83	CHODAN	Cultivar	1983	POL
57*	8816/79	CH-S	Cultivar	1979	POL
58	18,701/83	DAŃKOWSKIE NOWE	Cultivar	1983	POL
59	8820/79	DAŃKOWSKIE SREBRNE	Cultivar	1979	POL
60	7047/76	GARCZYŃSKIE LUDOWE	Cultivar	1976	POL
61*	18,639/83	GOLSKIE	Cultivar	1983	POL
62*	4266/74	KORTOWSKIE	Cultivar	1974	POL
63	7196/76	MIKULICKIE WCZESNE	Cultivar	1976	POL
64*	14,057/80	PANCERNE	Cultivar	1980	POL
65	7238/76	PULAWSKIE	Cultivar	1976	POL
66	7285/76	SZK 6B/65	Cultivar	1976	POL
67	18,657/83	TEMPO	Cultivar	1983	POL
68	7392/76	WŁOSZANOWSKIE NOWE	Cultivar	1976	POL
69	7404/76	BRIGODA DE MIRANDELA	Cultivar	1976	PRT
70	1393/71	PORTO	Cultivar	1971	PRT
71	6971/76	BRASOV 200-N	Cultivar	1976	ROM
72	8867/79	SUCEAVA 50	Cultivar	1979	ROM
73	5776/75	BURUNAJA	Cultivar	1975	RUS
74	2680/73	FALENSKAJA	Cultivar	1973	RUS
75	41/70	KAZANSKAJA	Cultivar	1970	RUS
76	14,917/80	KRUPNOZERNAJA	Cultivar	1980	RUS
77	2683/73	NOVOZYBKOVSKAJA 4	Cultivar	1973	RUS
78	45/70	SITNIKOVSKAJA	Cultivar	1970	RUS
79	14,969/80	SPASSKAJA MESTNAJA	Cultivar	1980	RUS
80	7172/76	WIR 7276	Landrace	1976	RUS
81	7449/76	87	Landrace	1976	SRB
82	8857/79	PONSI	Cultivar	1979	SWE
83	8869/79	SV. 6728	Breeding material	1979	SWE
84*	7076/76	1566	Landrace	1976	TUR
85*	7077/76	1794	Landrace	1976	TUR
86*	7079/76	2666	Landrace	1976	TUR
87	7084/76	3525	Landrace	1976	TUR
88*	7095/76	4018	Landrace	1976	TUR
89	7117/76	4317	Landrace	1976	TUR
90	2373/73	BEREGOVSKAJA	Cultivar	1973	UKR
91*	14,974/80	HARKOVSKAJA	Cultivar	1980	UKR
92*	6964/76	ATHENS ABRUZZI	Cultivar	1976	USA
93	14,156/80	DWARF WINTER	Cultivar	1980	USA
94	25,282/86	FREDERICK	Cultivar	1986	USA
95	7250/76	ROSEN	Cultivar	1976	USA
96	25,286/86	SCHNIDT	Cultivar	1986	USA
97	7287/76	TENNESSEE 4062	Breeding material	1976	USA
98	1306/71	WESER	Cultivar	1971	USA
99	14,059/80	BALBO	Cultivar	1980	ZAF
100*	7002/76	DL67/172	Cultivar	1976	ZAF

### DNA isolation

One hundred fifty seeds from each accession were treated with Funaben T (45% thiram, 20% carbendazim) and placed on Petri dishes on tissue paper at room temperature for germination. From the seedlings at the first leaf stage, fragments of about 1.5 cm were collected from the central part of the leaf blade. Each accession was represented by 6 bulk samples of 16 plants each. After harvesting, the material was frozen, freeze-dried (LABCONCO), and ground in a bead mill MM 100 (Retch). DNA was isolated from pooled samples using a Clean Plant PK DNA Purification Kit (CLEANNA) according to the manufacturer’s procedure. Concentration and purity of DNA were evaluated spectrophotometrically using NanoDrop 1000 (Thermo). Then, six pooled samples for each accession were combined in one in equal proportions and diluted to a working concentration of 20 ng/μl.

### SSR analysis

Seventeen SSR markers available in the public domain (Hackauf and Wehling 2002; Saal and Wricke 1999), representing all rye chromosomes, were included in the genetic diversity study (Table 2). Markers were selected according to previous studies by Targońska et al. (2016). The selection of markers was made on the presence of good quality and polymorphic products from a single locus. The markers were located on genetic maps (Bolibok-Brągoszewska et al. 2009; Hackauf and Wehling 2003; Milczarski et al. 2007; Stojałowski et al. 2009). One primer of each pair was labeled with one of the four fluorescent dyes: 6-FAM, VIC, NED, and PET. Multiplex PCRs were performed in a 10-μl volume containing approximately 20-ng template DNA, 1 pmol of each primer, and 5 μl AmpliTaq Gold™ 360 Master Mix (Life Technologies). Reactions were performed in an Arktk thermocycler (Life Technologies) with an initial denaturation step of 10 min at 94 °C, 10 cycles of 30 s at 94 °C, 1 min starting at 64 °C and decreasing 1 °C per cycle, 1 min at 72 °C followed by 35 cycles of 94 °C for 30 s, 55 °C for 45 s, and 72 °C for 1 min, and a final extension at 72 °C for 30 min. For PCR fragment size determinations, 0.25 μl of GeneScan600 LIZ Size Standard (Applied Biosystems) an internal size standard was mixed with 1 μl of diluted PCR product (1/10) and 9 μl formamide.

**Table 2:** SSR markers used in the study

Marker id	Chromosome	Reference	Multiplex	Products range (bp)	Dye
SCM009	1R	Saal and Wricke 1999	A	205–255	NED
SCM028	6R	Saal and Wricke 1999	A	128–130	VIC
SCM041	2R	Hackauf and Wehling 2002	A	131–160	FAM
SCM050	7R	Hackauf and Wehling 2002	A	98–145	PET
SCM063	7R	Hackauf and Wehling 2002	B	224–250	NED
SCM101	4R	Saal and Wricke 1999	B	150–200	FAM
SCM107	1R	Hackauf and Wehling 2002	A	232–252	FAM
SCM109	5R	Saal and Wricke 1999	B	127–145	PET
SCM112	3R	Hackauf and Wehling 2002	A	375–410	NED
SCM118	2R	Hackauf and Wehling 2002	A	145–166	VIC
SCM138	5R	Saal and Wricke 1999	B	102–128	FAM
SCM139	4R	Hackauf and Wehling 2002	A	126–142	NED
SCM152	5R	Hackauf and Wehling 2002	B	320–391	VIC
SCM155	4R	Hackauf and Wehling 2002	A	218–243	PET
SCM162	3R	Hackauf and Wehling 2002	B	128–197	VIC
SCM171	1R	Hackauf and Wehling 2002	B	216–223	PET
SCM180	6R	Saal and Wricke 1999	B	138–145	NED

The amplified products were analyzed by an ABI 3500 Genetic Analyzer (Applied Biosystems) using a 36-cm capillaries array filled with POP-7 polymer. The length of fragments was assessed against the size standard GeneScan™ 500 LIZ™ Dye Size Standard (Applied Biosystems).

### Evaluation of agronomic traits

Phenotyping has been carried out on rye plants growing on 1.5 m^2^ experimental plots. All observation and measurements have been carried out for three consecutive growing periods: 2015/2016; 2016/2017; and 2017/2018. Seeds were sown in autumn every 2–3 cm in rows 17.5 cm spaced. Before sowing, seeds were treated with seed dressing (Funaben T). During growing season, morphological characters as well as basic phenological data have been evaluated. Each observation has been carried out at the recommended time. Sowing, flowering, and wax maturity dates make a possible calculation of the length of vegetation and grain filling period. Plant emergence, winter hardiness, snow mold resistance, powdery mildew resistance, brown rust resistance, and stem rust resistance were expressed in a 1–9 point scale. Plant height, penultimate leaf length, and spike length were measured for 20 plants per plot and expressed in centimeters. The number of grains per spike was the average number for 20 manually harvested and threshed ears. One thousand grains of each accession were collected at random, weighed to record the seed index, and expressed in grams.

### Data analysis

#### Genotype data analysis

The length of the fragments was determined using the GeneMapper (Applied Biosystems) software. The amplified fragments from each accession were transformed into binary character matrix where the presence of a fragment of defined length was scored as 1, while its absence as 0. The obtained binary matrix was subjected to data analysis encompassing four key steps, i.e., evaluation of marker informativeness, genetic diversity analysis, genetic distance-based analysis, and population structure analysis.

At first, an assessment of the markers’ informativeness was carried out and it included the calculation of polymorphism information content (PIC) and resolving power (Rp) coefficients (Prevost and Wilkinson 1999; Roy et al. 2002).

In the next step, the binary matrix was subjected to genetic diversity analysis in which Nei’s unbiased coefficient of variation (uHe) and Shannon’s information index (*I*) were calculated (Brown and Weir 1983; Lynch and Milligan 1994). The significance of differences in coefficients in groups established based on the origin and biological status of an accession was assessed using ANOVA and post hoc Tukey’s HSD test.

The third step was to calculate the genetic distance using Jaccard’s coefficient and to perform hierarchical grouping with Ward’s method, principal coordinate analysis (PCoA), and hierarchical analysis of molecular variance (AMOVA) (Excoffier et al. 1992; Jaccard 1901).

The last stage of genotype data processing was to analyze the population structure using the Bayesian model-based analysis (Pritchard et al. 2000). The number of cluster (*K*) values was set from 1 to 15, with ten independent runs for each *K* (100,000 burn-ins and 500,000 iterations). An admixture model with correlated allele frequencies was employed. The CLUMPAK software was used to identify the number of real clusters in data (*K*). The optimal value of *K* was determined based on the *posteriori* probability of data for a given *K* and Δ*K* using a full search algorithm to obtain the best alignment of the cluster analysis (Evanno et al. 2005; Kopelman et al. 2015). Grouping based on model-based analysis was made by calculating Euclidean distance and performing Ward’s analysis. The differences in the proportion of clusters were tested using ANOVA and post hoc Tukey’s HSD test.

#### Phenotype data analysis

Two-way ANOVA analysis was carried out, based on 3-year field trial results, and post hoc Tukey’s HSD test was employed. The average values for the examined features were used as a basis for multiple factor analysis (MFA) to simultaneous analysis of qualitative and quantitative morphological traits. The averages for the traits were also standardized and a proximity matrix by the Gower coefficient was developed (Gower 1971).

#### Joint analysis

Genetic and phenotypic dissimilarity matrices were correlated using the Mantel test (Mantel 1967). A consensus configuration of both levels of describing rye collection was obtained by the generalized Procrustes analysis (GPA) (Gower 1975). Based on phenotypic and genotypic data, a core subset was selected from the whole tested accessions using the advanced M strategy implemented through a modified heuristic algorithm using the PowerCore (Kim et al. 2007).

#### Software

All the abovementioned data analyses were performed using the following software: Microsoft Excel 2016, XLSTAT Ecology (Addinsoft, Inc., Brooklyn, NY, USA), GenAlEx 6.501 (Peakall and Smouse 2006), and STRUCTURE 2.3.4 (Pritchard et al. 2000).

## Results

### Genotype analysis

#### Marker informativeness

Using a multiplex SSR technique, a total of 148 fragments were obtained of which 56.08% were polymorphic. The average number of fragments per marker was 8.7 and it was in the range 2–24 for SCM050 and SCM109 respectively. The participation of polymorphic fragments for each marker ranged from 0 (SCM050) to 80% (SCM118 and SCM028). The average value of PIC for all SSRs was 0.648. A multiplex A had a slightly higher PIC value (0.677) than a multiplex B (0.616). The maximum PIC was achieved by SCM101 (0.907), while the minimum value was found for SCM050 (0.00). The mean value of resolving power that describes the discriminatory potential of the markers was equal to 1.67 (multiplex A) and 2.54 (multiplex B). Detailed data is provided in Table 3.

**Table 3:** Marker statistics (*PIC*, polymorphism information content; *Rp*, resolving power coefficients)

Multiplex	Marker ID	No. fragments	Polymorphic fragments (%)	Unique fragments (%)	PIC	Rp
A	SCM009	6	67	17	0,762	2.34
	SCM028	5	80	0	0.774	1.54
	SCM041	11	45	55	0.824	2.04
	SCM050	2	0	0	0.000	0.02
	SCM107	5	20	40	0.593	0.62
	SCM112	7	57	29	0.641	1.8
	SCM118	15	80	20	0.809	4.96
	SCM139	4	75	0	0.304	1.4
	SCM155	6	17	67	0.709	0.3
Mean					0.677	1.67
B	SCM063	3	67	0	0.097	0.3
	SCM101	22	64	36	0.907	8.48
	SCM109	24	62.5	37.5	0.864	5.62
	SCM138	8	50	38	0.867	1.1
	SCM152	11	55	36	0.647	1.74
	SCM162	7	57	29	0.551	1.44
	SCM171	5	40	40	0.436	0.48
	SCM180	7	57	43	0.557	1.12
Mean					0.616	2.54
Total		8.7	52	29	0.648	2.08

#### Genetic diversity

Accessions were classified according to geographical region, country of origin, biological status, and date of acquisition. For such groups, the diversity coefficients were calculated, i.e., Nei’s unbiased coefficient of variation (uHe) and Shannon’s information index (*I*) (Fig. S1). The lowest variation was among the accessions originating from Finland (uHe = 0.191 and *I* = 0.257) and the highest between those originating from Germany (uHe = 0.266 and *I* = 0.385) and Turkey (uHe = 0.280 and *I* = 0.390). The analysis of variance (ANOVA) did not show significant differences in uHe and Shannon index values among accessions grouped by country of origin. There were no significant differences in the values of both coefficients between the groups separated based on continents from which the analyzed accessions were originating. Since as much as 73% of the surveyed accessions came from Europe, they were also labeled according to the European regions. ANOVA showed significant region-related differences in values of Nei’s unbiased coefficient of variation. The lowest variation was observed within the accessions from Central Europe and the highest within the South European. The values of the *I* coefficient did not show any significant differences between the regions of Europe. The results of the analysis following the biological status of accession showed that the highest diversity occurred within the group of landraces (0.246), significantly higher than the calculated for cultivars (0.187). Shannon’s index values did not show statistically significant differences in biological status. The date of acquisition of the accessions to the gene bank did not significantly affect the differentiation coefficients.

#### Clustering analysis

**Fig. 2:**
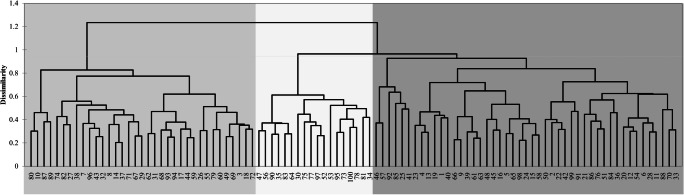
Hierarchical grouping by Ward's method performed based on the Jaccard’s distance matrix

**Fig. 3:**
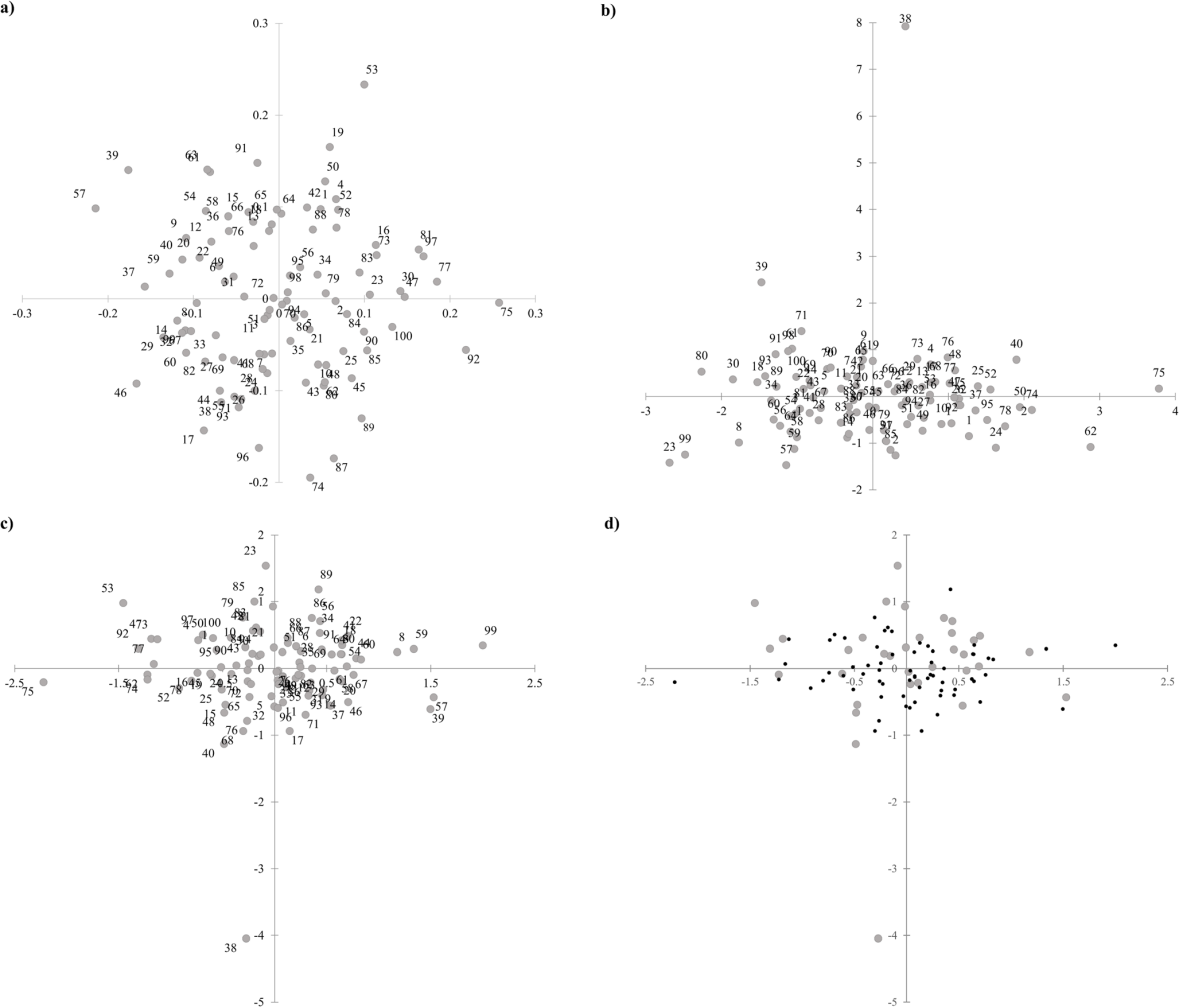
The plot of the first two axes of principal coordinate analysis of genotypic data (**a**); multifactor analysis of phenotypic data (**b**); generalized Procrustes analysis of genotypic and phenotypic data (**c**); generalized Procrustes analysis of genotypic and phenotypic data with indication of core collection (gray circles) (**d**). Accessions numbered in accordance with Table 1

The genetic distance calculated using Jaccard’s coefficient for 100 rye accessions ranged from 0.204 (“GALMA”–“PETKUSER MOORROGGEN”) to 0.710 (“ATHENS ABRUZZI”–“BALBO”). The average distance between accessions was 0.433. Hierarchical grouping by Ward’s method was performed based on the distance matrix. It showed the presence of three main clusters consisting of 35, 18, and 47 accessions. Within all clusters, there were lower-order structures. In the results of grouping, there were no links with the country of origin, geographical area, or biological status (Fig. 2). The first three main coordinates of PCoA explained in total only 16.5% of the whole variation. The graphs of the first and second main coordinates showed no the presence of separate groups, and the distribution of points in the two-dimensional space did not present a link with either the geographical region, country of origin, or biological status (Fig. 3a).

#### AMOVA

Analysis of molecular variance revealed no genetic structuring related to geographic regions. However, for the European accessions, 1.5% of variation in the genetic structure was correlated with five regions of Europe. In total, 3.4% of molecular variance originated from countries. Negative values for variation components indicated a lack of population structuring associated with the biological status and the year of acquisition (Table 4). Pairwise ɸPT for countries ranged from 0.042 (RUS-others) to 0.121 (CAN-POL) after excluding nonsignificant values. A total of 87.75% of the ɸPT values were insignificant at *p* = < 0.05. Ward’s grouping based on significant ɸPT values showed the presence of three groups composed of 5, 14, and 2 countries. Accessions from Germany, Austria, Romania, Czech Republic + former Czechoslovakia, and Poland formed the first group. The second group consisted of all other accessions except those from Spain and Turkey, which were classified in the third group (Fig. S2).

**Table 4:** Estimates of AMOVA results based on SSR markers for different grouping of 100 rye accessions

	ɸPT	*p*
Geographic regions	0.022	0.156
European regions	0.015	0.032
Countries	0.034	0.001
Biological status	− 0.01	0.929
Acquisition year	− 0.001	0.562

#### Population structure

**Fig. 4:**
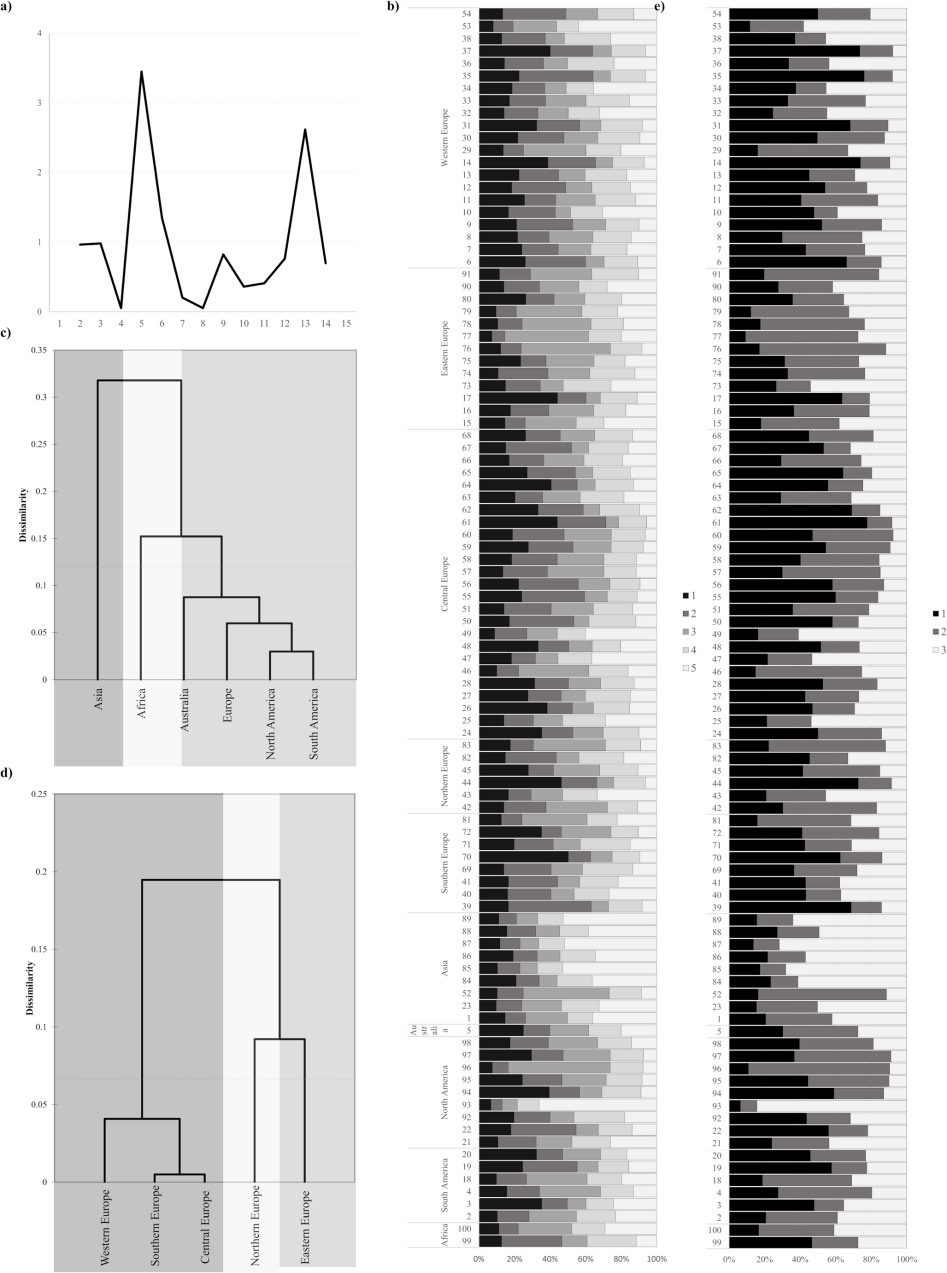
Inference of the population structure of rye accessions based on SSRs using a model-basedBayesian clustering carried out using Structure software (Pritchard et al. 2000) a) Results of the optimalsubpopulation model investigation by plotting ΔK of the data over ten runs, as implemented in Clumpaksoftware (Kopelman et al. 2015); b) Q-plot of genetic clusters assigned for K=5. Each color represents a uniquegenetic cluster. Each accession is represented by a horizontal bar with the colors showing the proportion of theindividual genotype derived from respective genetic clusters; c) Hierarchical grouping by Ward's methodperformed based on the Euclidean distance of Q values for k=5 for geographic regions; d) Hierarchical groupingby Ward's method performed based on the Euclidean distance of Q values for k=5 for European regions; e) Qplotof genetic clusters assigned for K=3

SSR dataset was implemented in STRUCTURE. The most probable number of clusters (*K*), evaluated using the ΔK method, indicated *K* = 5 sub-populations (Fig. 4a). However, the graphical representation of the results indicated the absence of any structuring in the examined materials accessions (Fig. 4b). No accession was strongly assigned (*Q* > 0.8) to an inferred population. The highest cluster membership coefficient (Q) values for five clusters were as follow 1–0.503 (“PORTO”); 2–0.467 (“FLORIDA BLACK WALLANCE SELECTION”); 3–0.573 (“SCHNIDT”); 4–0.282 (“BRIGODA DE MIRANDELA”); and 5–0.661 (“DWARF WINTER”). In geographical regions, the proportion of clusters was significantly different (*p* < 0.05) only for the fifth group. Its contribution was significantly higher in Asia than in Europe and both Americas. The differences in the proportion of all clusters were significant for countries. However, the HSD post hoc test distinguished homogeneous groups only for the third cluster. Its contribution was significantly higher in Iran than in Turkey, Belgium, and Belarus.

Dendrogram based on the results for *K* = 5 showed that the greatest similarity in the population structure was between North and South America, and the structure of Asian accessions formed a distinctive group (Fig. 4c). There were no statistically significant differences in population structure between European accessions. The Ward grouping showed a certain distinctiveness of the genetic structure of the accessions from central and southern and western Europe to those from eastern and northern Europe (Fig. 4d). In accordance with three main clusters detected as a result of grouping analysis and relatively high Δ*K*-value, a graphical display of results for *K* = 3 was also made (Fig. 4e). As before, a lack of a clear population structure and link to the place of origin or biological status was detected. Only one accession (“DWARF WINTER”) was assigned to the third group (*Q* = 0.84).

### Phenotype analysis

#### Variation analysis

The phenotypic evaluation included a total of 13 traits, i.e. six qualitative and seven quantitative ones. Due to the cross-pollination of rye and the absence of reproductive isolation, the statistical analysis could not include the seed index as a trait, which may be modified concerning the pollen donor genotype. A summary of the results obtained for the examined traits was presented in Table 5. Among the features tested, only the resistance to *Microdochium nivale* (Fr.) Samuels & I.C. Hallett, causing snow mold, was stable because none of the accessions showed symptoms of infestation. The highest variability (11%) occurred within the number of grains per spike. On average, the lowest number of grains per spike was obtained for “DWARF WINTER” (29.67) and the highest for “DOBRENICKE KRMNE” (61.67). The variability of the other traits ranged from 1 to 7% and it was reflected as insignificant in the analysis of variance (Table 6). A significant value of ANOVA output was calculated only for spike length and it was related to the country (*p* = 0.035) and region of origin (*p* = 0.013). The number of grains per spike was significantly higher for the accessions of Northern Europe than for those from Africa (*p* = 0.002). The other accessions formed a homogeneous group with an intermediate score. The variance analysis did not show significant differences between groups with different biological status or between individual accessions. On the other hand, all traits were significantly influenced by the climatic conditions in the years in which the field trials were carried out (*p* < 0.0001). Correlation analysis showed a moderate uphill relationship between two phenological traits i.e. grain filling period and the number of days to maturity (0.516). Plant height was moderately related to spike length (0.496) and the penultimate leaf length (0.393), as well as to winter hardiness (0.401). A weak uphill relationship was observed for stem rust resistance and grain filling period (0.261), spike length, and penultimate leaf length (0.240). A weak downhill correlation was found between stem rust resistance and spike length (− 0.303). In the remaining variants, the correlation coefficient was statistically insignificant (Table 7).

**Table 5:** List of phenotypic traits and their ranges. In brackets, the accession numbers according to Table 1 are shown

Trait	Unit	Minimum	Maximum	Average	Variation coefficient (%)
Emergence	Scale 1 (10%) – 9 (90%)	6.33 [38]	9 [47, 12, 98]	8.12	7
Winter hardiness	Scale 1 (10%) – 9 (90%)	6.33 [23, 79]	9 [75, 62]	8	7
Snow mold resistance	Scale 1 (very sensitive) – 9 (very resistance)	9	9	9	0
Powdery mildew resistance	Scale 1 (very sensitive) – 9 (very resistance)	7 [37]	9 [2, 23, 24, 32, 34, 39, 59, 61, 74, 87]	8.55	4
Brown rust resistance	Scale 1 (very sensitive) – 9 (very resistance)	6.33 [2]	8.83 [8, 26, 60, 83, 91, 98]	8.26	6
Stem rust resistance	Scale 1 (very sensitive) – 9 (very resistance)	8.33 [22]	9 [3, 5, 7, 9, 12, 18, 19, 20, 21, 28, 30, 31, 34, 37, 38, 47, 50, 56, 58, 61, 62, 63, 67, 68, 69, 70, 71, 80, 91, 93, 98, 99]	8.84	2
Grain filling period	Days	35 [24]	42 [61]	38.51	3
Days to maturity	Days	270.3 [97]	277.33 [80]	273.74	1
Plant height	cm	141.23 [57]	188.2 [47]	166.44	6
Penultimate leaf length	cm	20.93 [43]	28.57 [25]	23.88	5
Spike length	cm	9.87 [64]	14.87 [74]	11.71	7
Number of grains per spike	No.	29.67 [93]	61.67 [24]	50.53	11
Seed index*	g	25.97 [86]	40.27 [31]	33.95	8

**Table 6:** Results of ANOVA of phenotypic traits for different groupings of 100 rye accessions (*ns*, nonsignificant)

Trait	Genotype	Country of origin	Geographic region	Biological status	Year
Winter hardiness	ns	ns	ns	ns	< 0.0001
Snow mold resistance	*–*	*–*	*–*	*–*	–
Powdery mildew resistance	ns	ns	ns	ns	< 0.0001
Brown rust resistance	ns	ns	ns	ns	< 0.0001
Stem rust resistance	ns	ns	ns	ns	< 0.0001
Grain filling period	ns	ns	ns	ns	< 0.0001
Days to maturity	ns	ns	ns	ns	< 0.0001
Plant height	ns	ns	ns	ns	< 0.0001
Penultimate leaf length	ns	ns	ns	ns	< 0.0001
Spike length	ns	0.035	0.013	ns	< 0.0001
Number of grains per spike	ns	ns	0.002	ns	< 0.0001

**Table 7:** Correlation coefficient (*r*) between the average values of phenotypic traits (below diagonal) and significance level (below diagonal) (values in italics are different from 0 with a significance level alpha = 0.05)

Trait	Winter hardiness	Powdery mildew resistance	Brown rust resistance	Stem rust resistance	Grain filling period	Days to maturity	Plant height	Penultimate leaf length	Spike length	Number of grains per spike
Winter hardiness	–	ns	ns	ns	ns	ns	< 0.0001	ns	ns	ns
Powdery mildew resistance	− 0.175	–	ns	ns	ns	ns	ns	ns	ns	ns
Brown rust resistance	0.195	− 0.059	–	ns	ns	ns	ns	ns	ns	ns
Stem rust resistance	0.048	− 0.161	0.014	–	0.009	ns	ns	ns	ns	ns
Grain filling period	− 0.052	0.016	0.086	*0.261*	–	< 0.0001	ns	ns	ns	ns
Days to maturity	− 0.176	0.082	0.121	− 0.035	0.516	–	ns	ns	ns	ns
Plant height	*0.401*	− 0.087	0.134	− 0.158	− 0.049	− 0.103	–	< 0.0001	< 0.0001	ns
Penultimate length	0.164	− 0.175	0.159	− 0.076	− 0.052	0.007	*0.393*	–	0.016	ns
Spike length	− 0.031	0.166	− 0.095	− 0.303	− 0.162	0.021	*0.496*	*0.240*	–	ns
Number of grains per spike	0.153	0.141	0.137	0.111	− 0.101	0.082	0.012	0.023	0.185	–

#### Clustering analysis

The next step was to perform multiple factor analysis (MFA) which is used to analyze simultaneously various types of data (qualitative and quantitative). The first three eigenvalues corresponded to 18.7% of the total variance (7.12%, 6.03%, and 5.5%, respectively). The quantitative traits were highly related to the first axis while the qualitative data were correlated with the second one. Consensus plot of analysis results, i.e., MFA centroid, of the first and second coordinates showed a significant distinctiveness of “POLLUX” cultivar (Fig. 3b). It was related to its qualitative features. The accession was characterized by some of the worst winter hardiness. Separateness from the group of the studied accessions was also noted for cultivars “KAZANSKAJA” and “KORTOWSKIE” which demonstrated high resilience to all studied diseases and very good winter hardiness. Moreover, “FLORIDA BLACK WALLANCE SELECTION” stood also out of the group, due to weak winter hardiness and a low number of grains per spike. Neither the MFA centroids nor the qualitative or quantitative traits showed any link between the distribution of points in two-dimensional space and the country of origin, geographical region, and biological status.

### Joint analysis

#### Comparison and combination

The Mantel test performed for genotypic and phenotypic data indicated that the correlation between the matrices was very low (i.e., 0.091, *p* < 0.0001). Generalized Procrustes analysis (GPA) was carried out to minimize the scale effect and to achieve a consensus configuration based on genotypic and phenotypic data. Scaling was the most efficient transformation method (Table 8). The residuals by configuration after the transformation were equal indicating that both types of data matched the consensus configuration at a similar level. The consensus test pointed out that the configuration was authentic; however, it corresponded to low (0.153) proportion of the original variance. A scatter plot showed that the studied accessions formed a uniform group located in the center of the coordinate system (Fig. 3c). Significant autonomy was recorded for the cultivar “POLLUX” originating from Germany. Also, cultivars “KAZANSKAJA” from Russia, “BALBO” from South Africa, Polish “CH-S,” and “FLORIDA BLACK WALLANCE SELECTION” from the USA showed some distinctiveness from the central group. The GPA analysis showed no grouping pattern associated with the region or country of origin and the biological status of the sample. This outcome was not a surprise as no grouping pattern had been discovered previously for either genetic or phenotypic data.

**Table 8:** PANOVA table for individual stages of GPA on the data

Source	DF	Sum of squares	Mean squares	*F*	Pr > *F*
Residuals after scaling	2174	417.550	0.192		
Scaling	1	281.109	281.109	1463.611	< 0.0001
Residuals after rotation	2175	698.659	0.321		
Rotation	300	47.753	0.159	0.829	0.981
Residuals after translation	2475	746.412	0.302		
Translation	25	0.000	0.000	0.000	1.000
Corrected total	2500	746.412	0.299		

#### Core collection

Due to the low variation of phenotypic traits and the significant influence of climatic conditions on their appearance, only genetic data were used to establish the core collection. An advanced maximization strategy, performed through the modified heuristic algorithm, was assigned to select 34 accessions from 17 countries to form a core collection (Table 1). Among them, 20.6% were cultivars originated from Germany, 14.7% were Polish cultivars, and 11.8% were Turkish landraces. The representation of the accessions dedicated to the core collection can be viewed on the GPA chart (Fig. 3d).

## Discussion

In the presented study, the genetic diversity within *Secale cereale* ssp. *cereale* was analyzed using multiplex SSR fingerprinting and evaluation of major agronomic traits. The panel of 100 accessions, belonging to the collection of PASBG, included historical cultivars, landraces, and breeding materials from various geographical origins and represented the major portion of the intra-species genetic diversity that have been studied.

The efficiency of the used marker system was evaluated by calculating their PIC value. The mean value for the marker set was 0.648. This value was slightly higher than previously obtained by Targońska et al. (2016), where all of the SSR markers from the presented marker set were used; the PIC value calculated for SSR markers used for genotyping was relatively high, with an average of 0.57 (range 0.18–0.93), which indicates their high informativeness. The difference may result from the improvement status (mainly old cultivars vs. cultivars, landraces, and breeding materials), the population size (100 vs. 367), the number of analyzed markers, and the number of the unique fragments. Both the total number of fragments and the number of unique fragments in two experiments result also from the sensitivity of the methods of fragment separation and detection. Higher sensitivity of automatic capillary electrophoresis detection may have had an impact on the higher value of PIC in the presented experiment. The ability of primers to distinguish genotypes was also evaluated by calculating Rp. The mean value of the parameter was equal to 1.67 for multiplex A and 2.54 for multiplex B. The values of Rp were relatively high for SCM101 and SCM109. In the work of Rawat et al. (2014), the values of Rp calculated for SSR markers were lower than for ISSR and AFLP. The authors related it to the lower number of detected bands in comparison with other tested marker systems. Relatively low values of Rp, with mean = 2.37, were calculated also for SSR markers in diversity analysis of sugarcane (Hameed et al. 2012). Based on the above data and previous studies of Targońska et al. (2016) and Bolibok-Brągoszewska et al. (2014), it can be concluded that selected markers are suitable for genetic diversity analysis of rye germplasm.

The analysis of genetic differentiation of the collection of obsolete rye germplasm was performed using 16 polymorphic SSR markers evenly distributed in the genome. According to the literature data, the effectiveness of this type of analysis has been proved for 10–24 highly informative SSR markers in genus *Secale* (Akhavan et al. 2010; Boczkowska and Puchalski 2012; Jenabi et al. 2011; Maraci et al. 2018; Myśków et al. 2010; Targońska et al. 2016). However, it should be kept in mind that such a small number of analyzed loci can significantly limit the resolution of the performed analysis. This resulted in as much as 87.75% of statistically insignificant values for ɸPT pairwise comparisons for the examined accessions and had further implications for Ward’s grouping disorder that established a greater affinity between the Spanish accessions to Turkish landraces rather than any other European cultivars. However, the general consistency of presented here genetic diversity results with the previous study of Bolibok-Brągoszewska et al. (2014), involving 1054 polymorphic DArT markers, indicates that an error due to limited resolution did not significantly affect the overall merit of the study.

Both the results of the analysis of genetic distance and differentiation coefficients, i.e., Nei’s unbiased coefficient of variation and Shannon’s information index, indicated that the examined obsolete gene pool was relatively large. Taking into account that the analyzed accessions were mainly cultivars (population cultivars, landrace selection, and mutation cultivars), the obtained result indicates that the rye breeding programs conducted in the years 1960–1980 were based on initial materials with a relatively high degree of differentiation. It is generally believed that the continuous selection and crossing of closely related cultivars have led to a narrowing of the gene pool on which modern breeding is based (Plucknett and Smith 2014). This hypothesis seems to contradict the result acquired in our study. However, the results obtained by Bolibok-Brągoszewska et al. (2014) clearly indicated the narrowing of *Secale* gene pool along with domestication and breeding. The results obtained to date showed that the gene pool of modern breeding lines is significantly narrower than that of landraces and wild *Secale* species. Interestingly, the level of genetic similarity among modern cultivars and the obsolete ones preserved in the gene bank was quite similar albeit the principal coordinates analysis and population structure demonstrated the distinctiveness of these two gene pools (Bolibok-Brągoszewska et al. 2014). A similar pattern, i.e., a lower level, of variation of cultivars in relation to landraces was also obtained in the presented study. Notably, the variation of breeding materials ranks between landraces and cultivars. Perhaps this is due to the origin of several breeding programs, i.e., in Germany, Sweden, and the USA, in which various starting materials were used and the selection was carried out in different ways. Research carried out by Bolibok-Brągoszewska et al. (2014) confirmed also the expectation that obsolete cultivars show a higher affinity for landraces than modern cultivars and breeding lines. This suggests the use of a limited number of distinct sources of genetic variation in modern breeding.

It is noteworthy that the high genetic variation was not reflected in the phenotypic variability. The reason was probably that the SSRs revealed the diversity across the genome to a greater extent, while for phenotype, either the target genes are less diverse or are modified by the interactions between genotype and environment, which resulted in poor differentiation and low variability under field conditions.

Relatively high genetic variation was observed among accessions originating from Germany. According to the literature data, before the start of hybrid breeding, the population cultivars in this country represented one of the two gene pools, i.e., “PETKUS” or “CARSTEN” (Geiger and Miedaner 2009). However, among the German historical rye cultivars, there were also those originating from the landraces (Schlegel 2013). Thus, it may suggest the possibility of crossbreeding with cultivars or forms outside these two dominant gene pools. Furthermore, the level of variation of this accession group was influenced by the presence of cultivars obtained through mutagenesis, i.e., “DONAR” and “POLLUX” (Schlegel 2013). Despite the fact that they come from the same breeder, i.e., F. von Lochow and successor VVB Saat-und Pflanzgut, their genetic make-up is substantially different.

In the presented studies, the highest level of variation was found among landraces originating from Turkey. This result was in line with expectations (McCouch 2004) and also consistent with the previous extensive studies of large scale with the use of DArT (Bolibok-Brągoszewska et al. 2014) and SSR markers (Targońska et al. 2016) and small-scale (12 rye accessions) studies done using RAPD markers (Persson et al. 2001). Landraces are primitive, highly genetically heterogeneous populations that were originated in subsistence agriculture, so they give a relatively low but stable yield (McCouch 2004). Landraces displayed considerable diversity and were distant from accessions obtained by breeders. This finding is not surprising because landraces are closely related to the wild ancestors of rye and share with them much more variation than modern high-yielding cultivars (McCouch 2004). It is a well-known fact that in practical, modern rye breeding, genetic resources were under-utilized (Geiger and Miedaner 2009). The background for this was probably such drawbacks as strong adaptation to local conditions at the place of origin, significant differences between elite and obsolete and primitive germplasms for polygenic traits, intolerance to inbreeding and finally epistasis, and pleiotropy and linkages between desired and undesired alleles (Haussmann et al. 2004). The lack of use of landraces and old cultivars in modern breeding programs was also described for common oat (Boczkowska and Onyśk 2016). Taking into account that the variation level within a population of outcrossing, wind-pollinated species as rye is generally higher than between populations; it can be expected that old population cultivars and landraces may contain some genotypes or loci capable for improving quantitative traits that may be interesting for modern breeding (Boczkowska and Tarczyk 2013; DeVicente and Tanksley 1993; Gailīte et al. 2013; Hamrick and Godt 1989; Persson and Von Bothmer 2000).

The obtained results showed the lack of the genetic structure within the obsolete germplasm of rye. Hierarchical grouping by Ward’s method showed the presence of three clusters; however, no links between accessions within the subgroups of the country of origin, geographical area, or biological status were found. An only weak correlation was observed between the geographic origin of analyzed plant material and its genetic make-up. It may be the result of an intensive exchange of breeding and cultivation materials that have taken place between different parts of the world over the last 150 years. As a consequence, it is very difficult to detect the linkage between the genetic and geographical distance in crops (Boczkowska and Tarczyk 2013; Diederichsen 2008). However, in the case of minor crops not so intensively bred, the relationship with the region of origin is much more evident (Podyma et al. 2017; Podyma et al. 2019). Earlier analyses of rye also indicated a lack of a link between the genotype and place of origin. On the basis of organellar DNA sequences, Isik et al. (2007) clearly demonstrated that no genetic distinctiveness of the sample can be inferred from a distant place of origin alone. Similar results were obtained using DArT markers (Bolibok-Brągoszewska et al. 2014) and also by Chikmawati et al. (2012), Shang et al. (2006), and Persson and Von Bothmer (2000) through analyses of AFLPs, SSRs, and allozymes, respectively. Also important is the fact that rye pollen can disperse over large distances which can lead to spontaneous crossings (Kozumplik and Christie 1972).

It is noteworthy that even samples of the same rye cultivar “DOMINANT,” representing the same geographical origin, occupied two different positions in both the PCoA graph and the hierarchical grouping dendrogram. While this result could be attributed to a mistake in labeling of seed samples, another explanation may be found in the work of Chebotar et al. (2003), where it was suggested that for open-pollinated species, the genetic integrity of accession may be changed in each regeneration cycle, which can alter the SSR pattern. Particularly is that the accessions were not obtained directly from the breeder and have a different history. It is known that the fist one was transferred from the Institute of Agrobotany Tapioszele (Hungary) to Beltsville Agricultural Research Center: USDA ARS (USA) in 1963, and then to the PASBG (Poland) in 1976. The second one was transferred from Breeding Station Laski (Poland) to PASBG in 1971. Genetic changes in rye seeds induced by long-term storage effects and consecutive regeneration cycles were also identified by the use of AFLP markers (Chwedorzewska et al. 2002) and SSR markers (Boczkowska and Puchalski 2012). A confirmation of this thesis can be also found in the work of Targońska et al. (2016), where two samples of “DAŃKOWSKIE NOWE” and two of “DAŃKOWSKIE ZŁOTE” (one sample obtained directly from Polish breeding company Danko and the second from PASBG) showed different SSR patterns and, as a result, were clustered separately on PCoA graphs and dendrograms. In 1995, van Hintum and Knüpffer named such accessions as “common duplicates” that originate from the same initial accession but may have lost their genetic identity (allelic compositions) (Diederichsen 2009; van Hintum and Knupffer 1995).

Another goal of the study was the selection of accessions to form a core collection. Core collections are a set of accessions derived from an existing collection that are selected to represent the widest possible spectrum of genetic variation in a given population in order to minimize the cost of genetic conservation (Brown 1989). The selected core collection constituted 34 rye accessions. A limited number of accessions, characterized by high genetic variation, are a useful tool as a representation of the population in various studies. Core collections play also a very important role in gene banks which face significant problems connected with the size and organization of plant germplasm collections. Nowadays, seventy rye germplasm collections are maintained and the total number of accessions is estimated to be about twenty-seven thousand (FAO 2018b). The proposed core collection could be the first step to simplify access to genetic diversity contained in rye germplasm and to enable its efficient use in basic and applied research. Moreover, our core collection could be treated as a testing panel in evaluating newly developed genetic markers or in studies on sequence diversity of selected genome fragments.

According to FAO data, over the last 60 years, the world’s area under cultivation of rye has decreased more than seven times. However, on poor, light, and acidic soils in temperate climates, the rye still remains the most economical cereal. The progress made at the same time in breeding caused the yield of grain per hectare increased over twofold (FAO 2018a). Major objectives in rye breeding do not differ significantly from those for other cereals and these are yield, lodging resistance, dwarfness, tolerance to abiotic stress, and, in the case of hybrid cultivars, resistance to ergot and other pathogens is particularly important (Geiger and Miedaner 2009). Rye by nature is a cereal with lower susceptibility to disease than wheat or barley. However, it is infested by pathogenic fungi such as *Microdochium nivale* (Fr.) Samuels & I.C. Hallett (snow mold), *Oculimacula yallundae* (Wallwork & Spooner) Crous & W. Gams and *Oculimacula acuformis* (Boerema, R. Pieters & Hamers) Crous & W. Gams, (eyespot disease), *Gaeumannomyces graminis* (Sacc.) Arx & D.L. Olivier (take-all), *Blumeria graminis* (DC.) Speer (powdery mildew), *Rhynchosporium secalis* (Oudem.) Davis (*Rhynchosporium* leaf spots), *Puccinia recondita f*. Dietel & Holw. (brown rust, syn. leaf rust), *Puccinia graminis* Pers. (stem rust), and *Claviceps purpurea* (Fr.) Tul. (ergot). All the examined accessions showed high or very high resistance to four observed diseases i.e. snow mold, powdery mildew, and brown and stem rust. Brown rust is one of the most popular, airborne rye fungal diseases and is regularly observed throughout its cultivation range (Miedaner and Sperling 1995). In the past, brown rust–resistant genotypes were identified within gene banks collections and were found in cultivars, landraces, and wild species. Resistance was determined by the presence of one or two dominant genes (Schlegel 2013). Many different resistance genes have been identified (Klocke 2004; Roux et al. 2004; Roux et al. 2007; Wehling et al. 2003). Two cultivars “JOEGEWA-AUSLESE” and “KAZANSKAJA” showed total resistance to brown rust in the 2015/2016 season when the severity of the disease was the highest. Stem rust is a particularly acute problem in organic farming. In conventional cultivation, it is also difficult to control using fungicides. Although several qualitatively inherited genes have been identified that can be used in breeding, there are no resistant varieties so far. Earliness of cultivars helps to avoid serious yield losses (Schlegel 2013). In the 3-year cycle of the experiments, this pathogen manifested its presence to a very low degree, so it is quite difficult to determine the actual resistance of the tested set of accessions. Several genes with a high degree of inheritance for resistance to powdery mildew have been described so far. Both quantitative and qualitative heritability have been found. This pathogen is known for the rapid evolution of virulence and therefore, the discovery of a new resistance gene is an urgent necessity to improve the quality of wheat and, increasingly, rye (Schlegel 2013). The total resistance to powdery mildew was found both in cultivars such as “DEBRETT,” “DOBRENICKE KRMNE,” “FALENSKAJA,” “FLORIDA BLACK WALLANCE SELECTION,” “GOLSKIE,” “HESSDORFER JOHANNIS,” “JANOS,” “DAŃKOWSKIE SREBRNE,” and ‘SINGZHAU” as well as in landrace “3525” originated from Turkey. During the 3-year trial, the disease was present with different intensities in the examination fields. Snow mold is caused by soil-borne fungi, tolerant to low temperatures. It appears after mild winters with deep snow cover. Resistance to this disease is polygenic and during the selection process, heredity is at a low level (Schlegel 2013). The development of the disease can be controlled by seed treatment. During the evaluation of the tested set of accessions, the disease did not appear. This was facilitated by the weather conditions, i.e., very mild winters without snow cover. Resistance to the diseases was assessed only on the basis of naturally occurring infestation. To check the level of resistance to pathogens with varying degrees of virulence, more thorough laboratory tests are necessary. Field observations did not include such traits as yield or lodging because only the standard description of the accessions for the gene bank was made. Considering that the accessions under examination were rather tall, it should be assumed that they could tend to lodge. As earlier studies proved, lodging and plant height are correlated (Wegrzyn et al. 1996). However, the length of the culm is negatively correlated with the yield, because in the rye, the stem is the main organ of assimilation (Nalborczyk et al. 1981), so the breeding of extremely short-strawed rye cultivars does not result in high yields of grain. This is especially visible in conditions of severe stress.

## Conclusions

Based on the results of field observations, it is difficult to clearly specify accessions that could be attractive for breeders. In fact, the only strong premise is the relatively large size of the obsolete gene pool. Moreover, based on previous findings, it can be presumed that it is distinct from the gene pool of modern rye cultivars. The genetic distinctiveness of parents is a desirable feature, especially in hybrid breeding. It is also important that the absence or low phenotypic variability does not indicate a lack of genetic differentiation. This is important because the accessions in gene banks are still mainly characterized only by the description of morphological, phenological, and agronomic traits. This may result in a low level of interest among breeders in genetic resources, who do not see the potential of the genotype hidden under the uniform phenotype. This way, the resources stored in the gene bank in the hands of a skillful breeder, equipped with genomic selection tools, can become a valuable source of variability. The highest genetic variation of landraces originated in Turkey has indicated the direction of our further research that will be focused on material from the region of the species diversity center, which so far has not been characterized.

## Electronic supplementary material

ESM 1 (PDF 158 kb)

ESM 2 (PDF 107 kb)

## Data Availability

The data are available in the Open Science Framework (https://osf.io/wpcq9/) doi 10.17605/OSF.IO/WPCQ9.
