# Supplementary material for: Investigation of obsolete diversity of rye (Secale cereale L.) using multiplexed SSR fingerprinting and evaluation of agronomic traits
Source: J Appl Genet. 2020 Sep 7;61(4):513–29. doi: 10.1007/s13353-020-00579-z (PMC7652744; doi:10.1007/s13353-020-00579-z)
Supplement: Supplementary file 1 — (PDF 158 kb) [file 13353_2020_579_MOESM1_ESM.pdf]

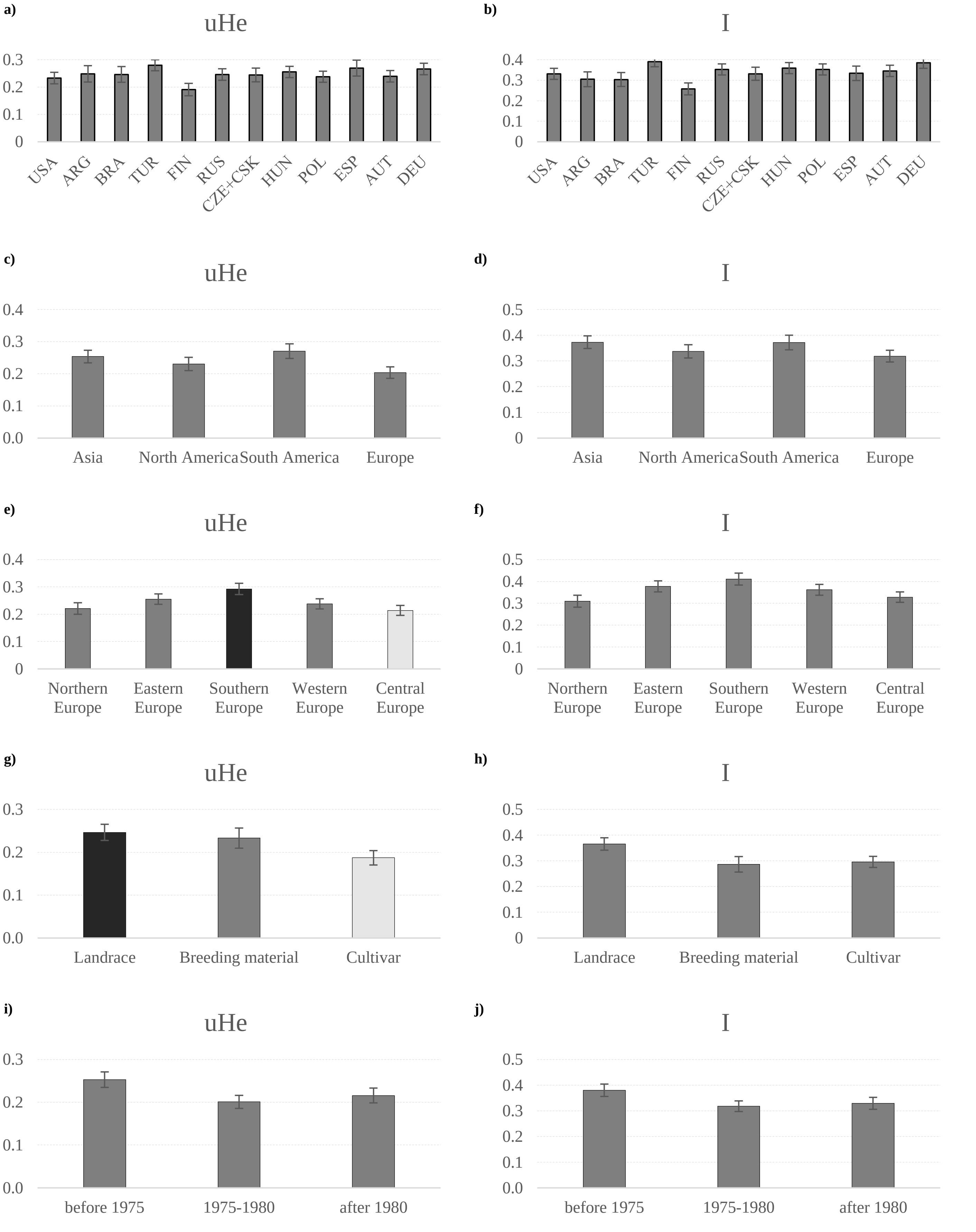

Figure S1. The diversity coefficients a) Nei's unbiased coefficient of variation (uHe) within countries; b) Shannon's Information Index (I) within countries; c) uHe within regions; d) I within regions; e) uHe within European regions; f) I within European regions; g) uHe within biological status groups; h) I within biological status groups; i) uHe within periods of accession acquisition; j) I within periods of accession acquisition. Homogeneous groups are color coded and the error bars illustrate the standard error.
