# Supplementary material for: Investigation of obsolete diversity of rye (Secale cereale L.) using multiplexed SSR fingerprinting and evaluation of agronomic traits
Source: J Appl Genet. 2020 Sep 7;61(4):513–29. doi: 10.1007/s13353-020-00579-z (PMC7652744; doi:10.1007/s13353-020-00579-z)
Supplement: Supplementary file 2 — (PDF 107 kb) [file 13353_2020_579_MOESM2_ESM.pdf]

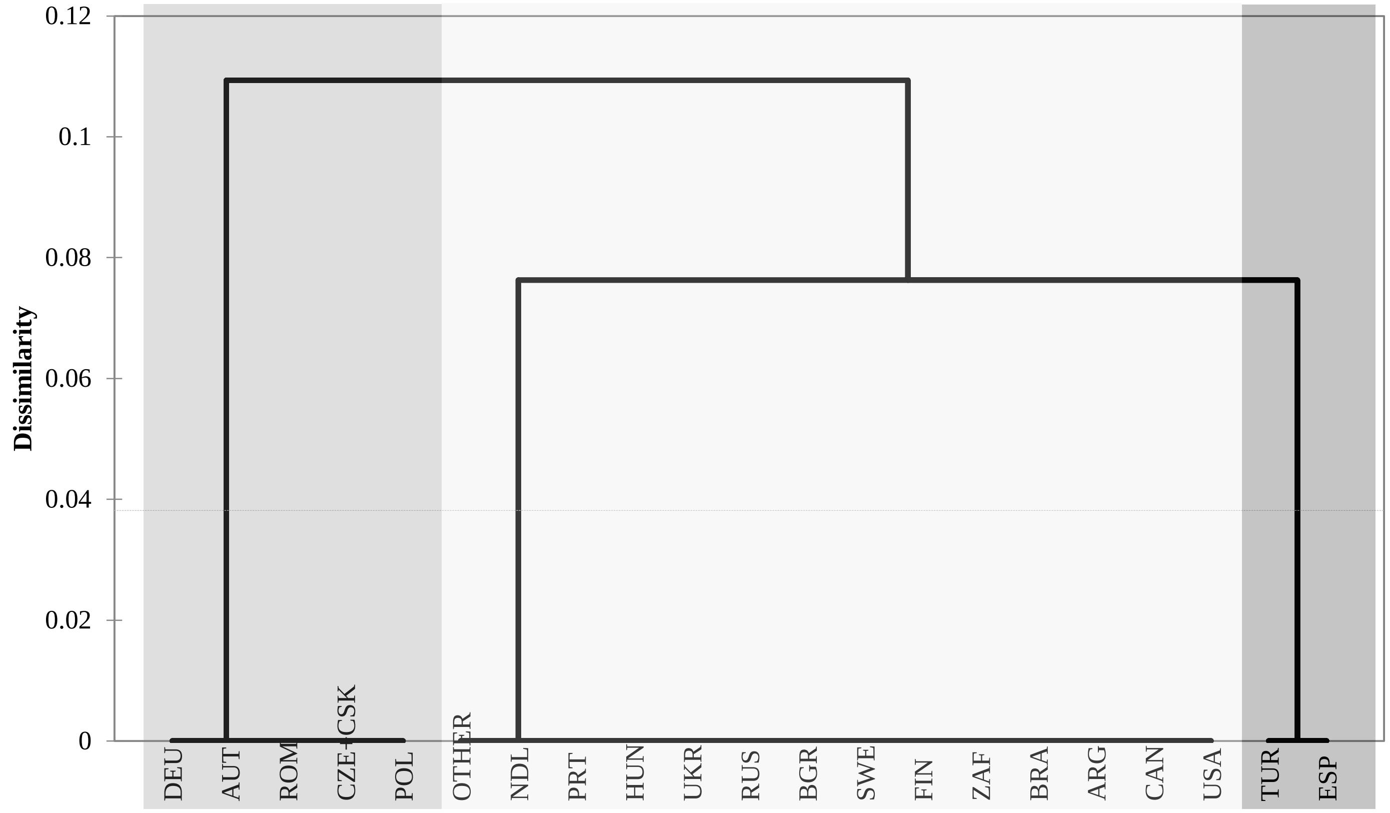

Figure S2. Hierarchical grouping by Ward's method performed based on significant pairwise ϕPT for countries.
